# Simple and Efficient Green Extraction of Steviol Glycosides from *Stevia rebaudiana* Leaves

**DOI:** 10.3390/foods8090402

**Published:** 2019-09-11

**Authors:** Verónica López-Carbón, Ana Sayago, Raúl González-Domínguez, Ángeles Fernández-Recamales

**Affiliations:** 1 Department of Chemistry, Faculty of Experimental Sciences, University of Huelva, 21007 Huelva, Spain; veronica.carbon@alu.uhu.es (V.L.-C.); ana.sayago@dqcm.uhu.es (A.S.); 2 International Campus of Excellence CeiA3, University of Huelva, 21007 Huelva, Spain

**Keywords:** steviol glycosides, green extraction, stevia leaves, stevioside, rebaudioside A

## Abstract

The food industry has currently shown great interest in alternative sweeteners to sugars with the aim of producing healthier products. In light of this, steviol glycosides are natural low-caloric sweeteners present in *Stevia rebaudiana*, which have additionally been described as bioactive components with potential therapeutic properties. In this work, a green method for the extraction of steviol glycosides from stevia leaves was optimized by applying a factorial screening design of five variables (time, temperature, agitation, grinding, and sample–solvent ratio) and the subsequent response surface design of Box-Behnken. The optimized extraction method allows for the recovery of stevia sweeteners in a simple and efficient manner by using tap water as the extractant, without the application of an auxiliary energy source to reduce costs, thus representing an interesting strategy for their industrial-scale production.

## 1. Introduction

The food industry has currently shown great interest in the use of low-caloric sweeteners instead of sugars and derivatives because of the consumer awareness with respect to their implication in the pathogenesis of diseases such as obesity and diabetes. Nowadays, the most marketed sweeteners are synthetic compounds (e.g., saccharin, acesulfame K, aspartame), but there is increasing controversy over their impact on health, so regulatory agencies have established maximum allowed contents to limit their potential toxicity problems. Thus, the use of natural sweeteners instead of artificial ones is emerging as an alternative with the aim of producing healthier foods. In this vein, the plant *Stevia rebaudiana* (Bertoni) Bertoni has been employed for centuries in South America due to its sweetening properties [1]. Stevia produces various glycosides derived from steviol (13-hydroxy-ent-kaur-16-en-19-oic acid), a diterpenic compound mainly found in leaves [2]. Among these steviol glycosides, also called steviosides, stevioside, and rebaudioside A, are the two most abundant and sweetest species (almost 300 times sweeter than sucrose), which account for 5–10% and 2–4% of the total weight of dry leaves, respectively. Rebaudioside A has the best flavor profile, while stevioside is responsible for the characteristic bitter aftertaste of stevia, sometimes reported as a ‘‘licorice’’ taste. Other minor steviol glycosides present in stevia leaves in lower concentrations are dulcoside A, steviolbioside, and rebaudiosides B-F [3]. In addition to their non-caloric sweetening properties, some studies have also shown that steviol glycosides, principally stevioside, could have therapeutic properties including anti-hyperglycemic, anti-hypertensive, anti-inflammatory, anti-tumor, anti-diarrheal, diuretic, and immunomodulatory effects [4,5,6,7,8]. 

The main obstacle for using natural sweeteners instead of artificial ones in the food industry is the higher cost associated with their extraction and subsequent characterization. Several analytical methodologies have been proposed for the extraction of steviol glycosides from leaves, usually based on the use of aqueous or alcoholic solvents, and optionally assisted by a source of auxiliary energy (e.g., ultrasounds, microwaves, liquid pressurization), followed by a clarification and purification step [9,10,11,12,13]. However, most of these procedures are time consuming and employ organic solvents, which are not applicable in the food industry. As an alternative, technologies based on membranes are being widely used nowadays for the recovery and purification of molecules with high commercial value in the pharmaceutical and food field. On the other hand, a wide variety of analytical strategies have also been proposed for the characterization of stevia extracts including nuclear magnetic resonance [14,15,16], Fourier transform infrared spectroscopy [17], capillary electrophoresis [18], and liquid chromatography. Among them, liquid chromatography, combined with ultraviolet-photodiode array detection (LC-DAD) or mass spectrometry (LC-MS), are the most commonly employed tools for the analysis of these compounds due to their precision, low cost, and wide range of applicability [17,19,20,21,22,23,24].

The aim of this work was to optimize a green procedure for the efficient extraction of steviol glycosides from stevia leaves, directly translatable to the food industry, and subsequent characterization of the resulting extracts by high performance liquid chromatography coupled to a diode-array detector (HPLC-DAD). 

## 2. Materials and Methods 

### 2.1. Reagents, Standards, and Samples

HPLC-grade acetonitrile was obtained from Fisher Scientific (Leicestershire, UK), while ultrapure water was obtained from a 109 Milli/Q water purification system (Millipore, Bedford, MA, USA). High-purity standards of stevioside and rebaudioside A were purchased from Sigma-Aldrich (Steinheim, Germany). Stock solutions (1000 mg L^−1^) were prepared in acetonitrile/water (8:2, *v*/*v*), and stored at 4 °C. Cellulose triacetate membranes (molecular cut-off 1 kDa) and the vacuum stirred system were supplied by Sartorius (Gotinga, Germany). The digital refractometer for Brix measurements was from Hanna Instruments (Woonsocket, RI, USA). Stevia dry leaves were kindly provided by Stevia Axarquia SL (Málaga, Spain).

### 2.2. Extraction of Steviosides: Experimental Design

To optimize the best extraction conditions for steviol glycosides, several factors such as grinding degree, temperature and time of extraction, agitation speed, and stevia leaf–solvent ratio were studied using an experimental design methodology. For this, the Brix degrees and content of stevioside and rebaudioside A were employed as dependent variables. First, a factorial screening design of five variables at two levels (2^5−1^) with V resolution was used to select factors significantly influencing the extraction process (by ANOVA, α = 0.05). The experimental levels and codes of variables investigated in the factorial design are shown in Table 1. Subsequently, the extraction factors that elicited significant effects on the dependent variables were optimized by applying the response surface design of Box-Behnken [25]. The relationship between the dependent and independent variables was fitted by a second-order polynomial model obtained by using the response surface methodology (RSM). Regression analyses were carried out according to Equation (1):

(1) $$Y\;={\;\beta }_{0}+\;\overset{3}{\underset{i=1}{\sum }}\beta_{i}x_{i}+\;\overset{3}{\underset{i=1}{\sum }}\beta_{\mathrm{ii}}x_{i}^{2}+\;\overset{3}{\underset{i,j=1}{\sum }}\beta_{\mathrm{ij}}x_{i}x_{j}$$

where Y is the dependent variable (i.e., Brix degrees, stevioside, and rebaudioside A contents); β_0_, β_i_, β_ii_, and β_ij_ are the regression coefficients in the intercept, linear, quadratic, and the interaction terms of the model, respectively, and x_i_ and x_j_ (i = 1, 3; j = 1, 3; i ≠ j) represent the non-coded independent variables. All experiments were performed in randomized order to avoid any systematic error.

Finally, a desirability function was applied to predict the unique optimum extraction conditions for all of the dependent variables. The desirability function is a transformation of each response variable to a corresponding desirability value (d_i_) between 0 and 1. Depending on whether a particular response is to be maximized, minimized, or assigned to a target value, different desirability functions can be used. The individual desirabilities are then combined by using the geometric mean to form the overall desirability (D). Thereby, the composite desirability function converts a multi-response problem into a single-response one.

### 2.3. Analysis of Steviosides

The content of steviol glycosides in the extracts was determined by high performance liquid chromatography coupled to a diode-array detector (HPLC-DAD) (Agilent Technologies, Santa Clara, CA, USA). Before analysis, extracts were tenfold diluted with water:acetonitrile (2:8, *v*/*v*) and filtered through nylon filters (0.22 μm pore size). Separations were performed on a hydrophilic interaction liquid chromatography (HILIC) column (50 × 2.1 mm, 2.6 μm) from Phenomenex (Torrance, CA, USA). Pure water and acetonitrile were delivered as aqueous (A) and organic (B) mobile phases, according to the following gradient program: 0–3 min, 90% B; 3–4 min, 90–80% B; 4–6 min, 80–70% B; 6–8 min, 70–90% B; 8–14 min, 90% B. The flow rate was set at 0.2 mL min^−1^, the column was thermostated at 25 °C, and the injection volume was set at 3 μL. The diode-array detector operated in the range from 190 to 350 nm. Steviol glycosides were identified by comparing the retention times and UV–Visible spectra with those from the reference standards. Quantification was carried out by external calibration using data acquired at 210 nm.

### 2.4. Method Validation

The analytical method was fully validated according to the UNE 82009-2:1998 normative. Calibration curves were prepared in water:acetonitrile (2:8, *v*/*v*) at seven concentration levels in the range 1–200 mg L^−1^ and 1–160 mg L^−1^ for stevioside and rebaudioside A, respectively, by diluting individual stock solutions of standards. The linearity of the method was evaluated by analysis of variance to verify the fitness of the straight-line model by means of the F-test [26]. The limits of detection (LODs) were estimated as the lowest concentration, giving an average signal-to-noise (S/N) ratio above 3. The repeatability (within-day precision) and reproducibility (day-to-day precision) were assessed by analyzing six extracts within the same day and at 5-day intervals over a period of one month in duplicate, respectively.

### 2.5. Statistical Analysis

All statistical analyses were performed using STATISTICA 7.0 software (StatSoft, Tulsa, OK, USA). The quality of fit of regression models was expressed by the coefficient of determination (R^2^ and R^2^_adj_), and its statistical significance was determined using the *p*-value by testing for lack of fit. Analysis of variance (ANOVA) with 95% confidence level was carried out for each response variable and the significance of each coefficient was determined by using the F-test. The three-dimensional surface plots and contour plots were drawn from regression models to highlight the effects of the independent variables on response variables. To predict the unique optimal extraction conditions valid for all of the dependent variables, the desirability function method was employed.

## 3. Results and Discussion

The main aim of this work was to optimize a simple and efficient extraction method for recovering steviol glycosides from stevia leaves. To facilitate its implementation in the food industry, we discarded the use of organic solvents not compatible with the dietary use of the resulting extracts, and avoided the application of auxiliary energy sources (e.g., microwaves, ultrasounds) to reduce costs. Thus, tap water was employed as the extracting solvent, followed by membrane filtration for preparing steviol glycoside-rich extracts, which is perfectly compatible with the principles of green chemistry. In this study, five extraction factors were investigated with the aim of maximizing the extraction efficiency of steviol glycosides from stevia leaves including the total extraction time, temperature, and agitation applied during extraction as well as the state of leaves grinding and sample-to-solvent ratio. To optimize the best extraction conditions, the extracts were subjected to refractometry to measure the Brix degrees and to HPLC-DAD analysis to determine the content of the major steviol glycosides of stevioside and rebaudioside A. Preliminary experiments were conducted to select the optimum separation conditions, and the best results in terms of chromatographic retention, peak shapes, and reproducibility were obtained by using hydrophilic interaction chromatography (HILIC), in agreement with the findings reported by Woelwer-Rieck et al. [19]. This method was then fully validated according to the UNE 82009-2:1998 normative. The linearity of the method was excellent for both steviol glycosides, with correlation coefficients (R^2^) higher than 0.99 and percentages of linearity (%L) equal to 99.3 and 98.6%, respectively. The sensitivity of the method was estimated by computing the limits of detection for stevioside (LOD = 1.39 ppm) and rebaudioside A (LOD = 1.46 ppm). These results were satisfactory for quantitating steviol glycosides at concentrations usually found in stevia leaf extracts. Relative standard deviations calculated from five-time repeated injections were below 5%, thus demonstrating the excellent instrumental precision.

### 3.1. Selection of Factors Affecting the Extraction Efficiency: Factorial Screening Design

As a first step, we applied a factorial screening design (2^5−1^) to determine which factors (time, temperature, agitation, grinding, and sample-solvent ratio) had a significant influence on the extraction efficiency. Each factor was studied at two levels, which were coded so that their values ranged between −1 and +1, resulting in a 16-run experimental matrix, as shown in Table 1. The content of stevioside, rebaudioside A, and Brix degrees of extracts were employed as dependent variables, and were subjected to analysis of variance to evaluate the significance of each factor. As can be seen in Table 2, the extraction time and agitation did not show any significant effect on the dependent variables studied. On the other hand, the sample-to-solvent ratio had strong positive effects on the three response variables, while the degree of grinding and extraction temperature positively influenced the content of stevioside and Brix degrees, respectively. It should be noted that these findings are in agreement with those reported for the extraction of other bioactive compounds such as polyphenols [27,28].

### 3.2. Response Surface Methodology for the Selection of Optimum Extraction Conditions

Based on previous results, the sample-to-solvent ratio, grinding degree, and temperature of extraction were subsequently optimized by means of the response surface methodology (RSM). The other variables (i.e., time and agitation) were kept at levels providing the best recoveries in the screening analysis: 20 min of extraction without agitation, in line with the results reported by Lorenzo et al. [21] and Jentzer et al. [29]. The RSM is a compilation of statistical and mathematical techniques based on the fit of empirical models to the experimental data, and the exploration of experimental conditions until their optimization [30]. To reach this goal, linear or square polynomial functions are employed to describe the system under study and to find the levels of variables that produce desirable values on the responses. This methodology is more favorable than traditional single parameter optimization, since it reduces time, space, and raw material usage [31]. In this work, a Box-Behnken experimental (BBD) design with 15 runs and three replications at the central points was used to define the optimal extraction conditions. Experimental values for response variables (i.e., stevioside, rebaudioside A, and Brix degrees) obtained from the BBD are listed in Table 3. These results were then fitted to a second-order polynomial model Equation (1), and the corresponding regression coefficients describing the quantitative relationship between dependent variables and factors are shown in Table 4. The statistical validity of these models was assessed by ANOVA through the regression and lack of fit F-tests. Briefly, a model is considered well fitted to the experimental data if it presents a significant regression and a non-significant lack of fit [31]. In both cases, a F value was calculated by means of a quotient of mean squares due to each of the sources of variation (regression, residual, lack of adjustment, and pure experimental error), and was then compared with the Fisher distribution, taking into account the corresponding freedom degrees associated with each variance. To check the significance of the regression, the ratio between the mean square of the regression and the mean square of the residuals was calculated, so that if this value was higher than the tabulated F value, the mathematical model would be well fitted to the experimental data. On the other hand, the ratio between the mean square due to the lack of fit and the mean square due to the pure experimental error was calculated to test the lack of fit. If this value was greater than the tabulated F value, there is evidence of the lack of adjustment and the model must be improved. Additionally, the coefficient of determination (R^2^) and adjusted determination coefficient (R^2^_adj_) were also used to provide additional confirmation of the statistical validity of the model fit. R^2^ is an estimate of the fraction of the overall variation in the data explained by the model, and R^2^_adj_ shows the relationship between the experimental data and the fitted model. Thereby, greater correlation between the observed and predicted values are obtained when these coefficients are close to the unit. 

According to the results from the Fisher tests, the contents of stevioside and rebaudioside A were significantly affected by the sample–solvent ratio (linear effect), grinding (quadratic effect), and the interaction between temperature and grinding (Table 4). Furthermore, a quadratic effect of the sample–solvent ratio was also found for stevioside. For the Brix degrees responses, all factors were significant with the exception of the linear effect of the sample-to-solvent ratio, being the linear term of grinding the variable with the largest effect. Regarding stevioside content, the largest effects were elicited by the quadratic term of grinding, followed by the linear and quadratic terms of the sample-to-solvent ratio. The quadratic term of grinding produced the largest effect on rebaudioside A content, followed by the linear term of the sample-to-solvent ratio. Models built for stevioside and rebaudioside A showed statistical valid fitness (*p* < 0.05 for the regression F-test) and non-significant lack of fit (*p* > 0.05). In addition, the coefficients of determination were estimated as 0.986 and 0.961 for stevioside and rebaudioside A, respectively, indicating that only less of 5% of the total variation could not be explained by the models. Furthermore, the adjusted coefficients of determination were very close to the corresponding R^2^ values (0.962 and 0.891 for stevioside and rebaudioside A, respectively), providing further evidence that the fitted multiple linear model has acceptable validity. However, the model for Brix degrees was not successfully validated (*p* > 0.05 for model, *p* < 0.05 for lack of fit), only accounting for 78.2% of the variability in the response.

**Figure 1:**
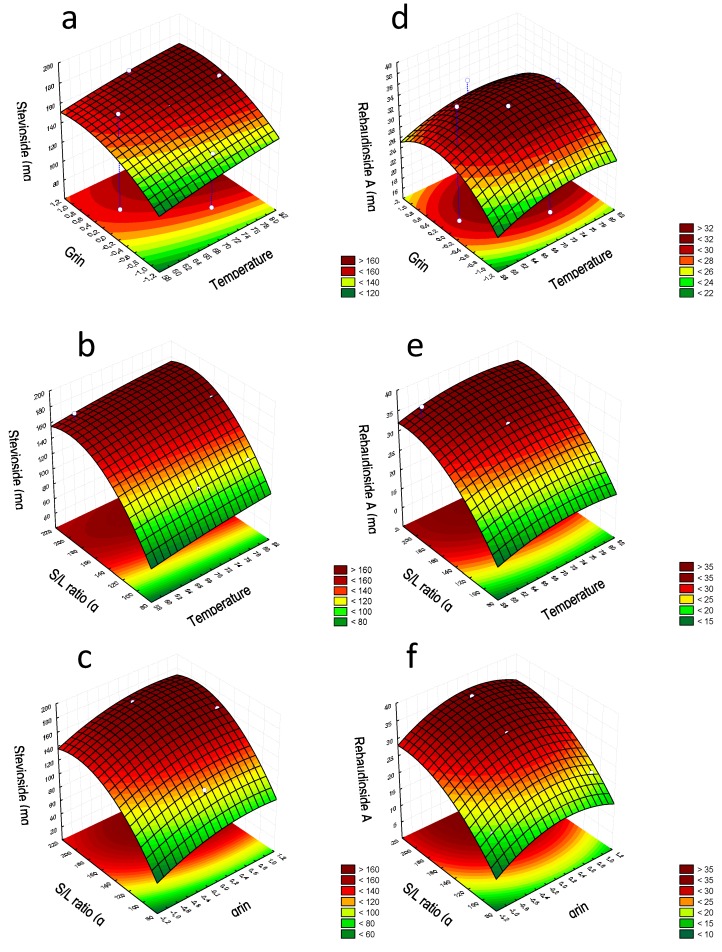
Response surface plots showing the effects of factors on Stevioside (**a**–**c**) and rebaudioside A (**d**–**f**) concentrations.

Finally, the regression models generated for stevioside and rebaudioside A concentrations were used to find the optimal value for the three extraction factors investigated: degree of grinding, temperature, and sample-to-solvent ratio. For this purpose, the relationship between independent and dependent variables was visualized in tri-dimensional representations of the response surfaces and bi-dimensional contour plots (Figure 1). These plots were obtained depicting two variables within the experimental range and keeping the third variable constant at the zero level (coded value corresponding to the central point of the experimental domain). As can be seen, the most dominant factor influencing the extraction of stevioside was the grinding of leaves. This factor had a quadratic effect with improved extraction of stevioside, while increasing the grinding until reaching a maximum level, after which a slight decrease was observed (Figure 1a). The sample-to-solvent ratio also caused a quadratic effect on the stevioside extraction similar to that observed for grinding, while the effect of temperature was linear, regardless of the grinding and S/L sample-to-solvent (Figure 1b,c). Using this model, the optimal extraction conditions for the stevioside were set as follows: temperature 99 °C, intermediate grinding level, and sample-to-solvent ratio 192. Under these conditions, the maximum extraction calculated using the RSM was 188.64 mg L^−1^. The effects of the experimental factors on rebaudioside A concentration were similar to those observed for stevioside (Figure 1d–f). In this case, the optimal conditions were: temperature 74.6 °C, very low grinding degree, and sample-to-solvent ratio 209 (maximum concentration calculated by RSM 36.12 mg L^−1^). 

To simultaneously maximize the extraction of both steviol glycosides, a desirability function approach was finally applied. This method is based on transforming each response variable to give a desirability value that is proportional to the priority given to the response variable. After applying the desirability function method for the stevioside and rebaudioside A concentrations, it can be concluded that 200 g L^−1^ for leaf–water ratio, intermediate degree of grinding, and 75 °C were the optimal conditions for the extraction of steviol glycosides from stevia leaves. These extraction conditions are very similar to those reported by Das et al., who tested the efficiency of hot water as a green solvent for the extraction of rebaudioside A from stevia leaves [10]. It should be noted that, while in this previous study the authors evaluated the recovery of rebaudioside A, the response variable considered in the present work was the content of steviol glycosides in the extract. The leaf–water ratio employed by Das et al. was significantly lower (2.36% vs. 200 g L^−1^), but they employed de-ionized water instead of tap water to perform the extraction, the optimized extraction time was significantly higher (51 min vs. 20 min), and they applied continuous stirring during the process, thus evidencing the higher simplicity of the extraction method described in the present study. 

## 4. Conclusions

According to the literature, there is a wide variety of extraction techniques for the highly efficient isolation of steviol glycosides from stevia leaves [13]. However, most of these techniques require specific equipment and/or costly working conditions. In this work, we applied a factorial screening design of five variables and a subsequent Box-Behnken response surface design to optimize the extraction of steviol glycosides from stevia leaves by using hot tap water as the extractant and without the application of any auxiliary energy source. The optimum extraction conditions were as follows: extraction time, 20 min; leaf–water ratio, 200 g L^−1^; temperature, 75 °C; by applying an intermediate degree of grinding and without agitation. The method proposed in this work is easy to apply, with low cost, and is environmentally friendly, thus providing extraction efficiencies comparable to results obtained by other authors [9,10,11]. 

## Figures and Tables

**Table 1:** Experimental levels and codes for independent variables investigated in the factorial screening design.

Independent Variables (Factors)	Codes	Levels	
		−1	+1
Temperature (°C)	X1	37	100
Sample-to solvent ratio (g L^−1^)	X2	6.25 (12.5 g in 2 L)	12.5 (25 g in 2 L)
Grinding	X3	without grinding	with grinding
Agitation	X4	without agitation	with agitation
Time (min)	X5	10	60

S: sliced; W: without grinding; G: with grinding.

**Table 2:** Analysis of variance (*p*-value) for the dependent variables considered in the factorial screening design.

Factors	Brix Degrees	Stevioside (ppm)	Rebaudioside A (ppm)
Temperature	0.002696	0.083793	0.047323
Sample-to solvent ratio	0.000187	0.000607	0.000642
Grinding	0.045356	0.008628	0.037213
Agitation	0.114666	0.047176	0.096332
Time	0.365637	0.457476	0.980448

**Table 3:** Experimental design runs for Box-Behnken design and experimental values of responses.

Order	Independent Factor						Responses		
	X1		X2		X3		Brix Degrees	Stevioside (ppm)	Rebaudioside A (ppm)
1	60	−1	100	−1	S	0	2.50	81.13	15.63
2	80	1	100	−1	S	0	2.75	120.53	23.40
3	60	−1	200	1	S	0	3.10	178.47	37.25
4	80	1	200	1	S	0	4.05	171.07	33.77
5	60	−1	150	0	W	−1	2.70	127.22	23.57
6	80	1	150	0	W	−1	4.60	139.55	26.48
7	60	−1	150	0	G	1	3.25	153.99	26.68
8	80	1	150	0	G	1	3.65	170.65	29.70
9	70	0	100	−1	W	−1	2.45	88.82	18.33
10	70	0	200	1	W	−1	3.50	144.05	27.89
11	70	0	100	−1	G	1	4.10	114.54	21.77
12	70	0	200	1	G	1	4.20	172.24	32.93
13	70	0	150	0	S	0	3.55	155.82	30.65
14	70	0	150	0	S	0	3.45	157.74	33.09
15	70	0	150	0	S	0	3.50	161.45	31.24

S: sliced; W: without grinding; G: with grinding.

**Table 4:** Regression coefficients and analysis of variance of the model obtained by response surface methodology (RSM).

Regression Coefficients	Responses		
	Brix Degrees	Stevioside	Rebaudioside A
Β_0_	−11.5583	−475.418	−148.842
Β_1_	0.3583	6.093	2.911
Β_2_	0.0055	4.599	0.857
Β_3_	2.9750	4.547	0.462
Β_12_	0.0004	−0.023	−0.006
Β_13_	−0.0300	0.108	0.003
Β_23_	−0.0040	0.012	0.008
Β_11_	−0.0027	−0.013	−0.014
Β_22_	−0.0001	−0.008	−0.001
Β_33_	0.2833	−9.185	−3.664
**Validation of the model**			
R^2^	0.782	0.986	0.961
R^2^_adj_	0.391	0.962	0.891
*p*-value (model)	0.230361	0.000374	0.005032
*p*-value (lack of fit)	0.0075	0.1395	0.2334
